# Simple and robust method for determination of laser fluence thresholds for material modifications: an extension of Liu’s approach to imperfect beams

**DOI:** 10.12688/openreseurope.13073.2

**Published:** 2021-06-25

**Authors:** Mario Garcia-Lechuga, David Grojo

**Affiliations:** 1 Aix Marseille Université, CNRS, LP3, UMR7341, Marseille, 13288, France; 2 Departamento de Física Aplicada, Universidad Autónoma de Madrid, Madrid, 28049, Spain

**Keywords:** Laser damage, material ablation, laser-induced material modification, beam metrology, damage threshold, ablation threshold, ultrafast laser, laser machining, laser beam effects

## Abstract

The so-called D-squared or Liu’s method is an extensively applied approach to determine the irradiation fluence thresholds for laser-induced damage or modification of materials. However, one of the assumptions behind the method is the use of an ideal spatial Gaussian beam that can lead in practice to significant errors depending on beam imperfections. In this work, we rigorously calculate the bias corrections required when applying the same method to Airy-disk like profiles. Those profiles are readily produced from any beam by insertion of an aperture in the optical path. Thus, the correction method gives a robust solution for exact threshold determination without any added technical complications as for instance advanced control or metrology of the beam. Illustrated by two case-studies, the approach holds potential to solve the strong discrepancies existing between the laser-induced damage thresholds reported in the literature.  It provides also an appropriate tool for new studies with the most extreme laser radiations.

## Plain language summary

Since the invention of the laser, the determination of optical damage and material modification thresholds is key for various applications from high-power laser developments, laser micromachining to laser nanosurgery. However, it is striking to note that strong discrepancies still persist between the reported thresholds for apparently similar conditions. This holds also for femtosecond laser dielectric ablation for which the usually admitted very strict threshold response should prevent any ambiguity. In this paper, we investigate the potential errors inherent to the standard metrology due to Gaussian beam imperfections. From this analysis, we propose a modified method for improved precision without technical complication. The key is to rely on well-defined Airy disk beams that can be readily obtained in any experimental configuration. Combined with biased-corrections that can be rigorously calculated for these beams, this solves reliability and robustness issues. The validity and performance of this simple approach is confirmed by femtosecond laser ablation measurements. The method opens the way for new studies with the most extreme laser radiations that suffer from complex metrologies. 

## 1. Introduction

The determination of the local laser fluence (or intensity) is critical when working with ultrashort laser pulses, since this marks the onset for risks in front of laser exposure (skin or corneal damage) ^1– 3^ or damage of optical materials ^4– 9^. However, the fluence is not a directly accessible quantity, as for its determination it is necessary to characterize both the integrated pulse energy and its spatial distribution. Although the energy is easily measurable by calibrated photodiodes, thermal sensors or pyroelectrics sensors, the determination of the spatial beam distribution can be more complex depending on the considered radiation and the precision needed.

The most precise method for beam profiling is obviously by direct imaging. The use of 2D-sensors or cameras have been applied for this purpose. This methodology is of easy application for collimated beams with size comparable to the camera array. Moreover, this methodology is also applied for focused beams by introducing an optical system (normally a microscope objective and a tube lens) for re-imaging and magnifying the small laser spot onto the camera detector ^10– 13^. However, there are two limitations associated to this approach: (i) It is experimentally difficult to design and implement a perfect imaging system that will not distort the observation. (ii) The spectral response range of cameras is limited with our current technology. For high-resolution silicon technologies, this basically limits the applicability to the visible or near-infrared domain of the spectrum. Out of this range, different technologies exist (including InGaAs for the extended near-infrared) but they are not routinely available in laboratories and often costly despite more limited performances (pixel size and dynamic range).

Alternatively, there are strategies to retrieve characteristics of the beam shape without the need of imaging it. The first set of techniques are the ones using an obstacle to partially block the beam (knife-edge ^14, 15^ or a wire ^16^), measuring the transmitted energy and retrieving by algorithmic calculations the beam waist. A second set of techniques are the so-called impact-based strategies. Those are of particular interest since the strict threshold response of material to ultrashort pulse irradiations makes that induced modifications can be taken as direct imprints of the laser profile ^13^. This strategy analyses the shape of modifications (ablation, changes of reflectivity, etc) produced at different pulse energies, associating the modification borders to a local fluence corresponding to the fluence threshold for modification. As an example, this strategy has been effectively applied for characterising the spatial distribution of ultrashort X-rays pulses ^17, 18^, that is in a particularly challenging spectral range for direct imaging technologies.

Among the impact-based strategies, there is a technique that stands out for its simplicity: the Liu’s method ^19^. Assuming a threshold modification response, this method allows to the user to retrieve the waist of a Gaussian beam by a linear fit when representing the diameter (square) of the modification versus the pulse energy (in logarithmic scale). This method published in 1982 is still extensively used in the ultrafast laser community, an aspect that can be illustrated by more than 600 citations since 2015 (for a total of around 1050 citations, data extracted from “ISI Web of Science”). The success of this technique is not only because the beam waist becomes easily accessible, but also because the modification fluence threshold of any material can be obtained by using it. This second potentiality was not commented on in the original paper, but rapidly had an impact according to evidence of its exploitation only a few years later ^20, 21^.

Even though Liu’s method is very frequently used, it is not always applied correctly since it provides only accurate fluence analysis if the irradiation beam is perfectly Gaussian (the assumption of the method). This condition cannot always be fulfilled as beam imperfections from laser systems or practical optical set-ups (e.g. aberrations) often occur. Therefore, the applicability of Liu’s method with unperfect beams (asymmetries, pedestals, etc) could be one of the reasons on the large dispersion of the ablation fluence threshold values reported on test materials (as fused silica) for apparently similar conditions ^5^. This raises an important issue that can be summarized with the following circular reasoning. How can we trust the fluence values obtained by Liu’s method if we do not know if the beam is perfectly Gaussian? How can we certify having a Gaussian beam if what we wanted with Liu’s method was to avoid beam imaging?

In this article, we present an extension to the Liu’s method to make it valid for beams that clearly deviate from the Gaussian approximation. This extension relies on exact correction factors to account for Liu’s method results when irradiating with a beam with Airy disk-like shape. This close-by Gaussian spot is a characteristic diffraction profile that can be directly generated by introducing a circular aperture in the beam path before the focusing element, a strategy commonly used in optical set-ups for laser material processing ^22– 26^.

This article is structured as follows. In the second section, a complete explanation of the original Liu’s method is presented. In the third section, the calculations of the correction factors to be applied on the Liu’s method when irradiating with a perfect Airy-disk are shown. In the fourth section, we repeat the calculation for more realistic cases, using truncated beams generated by different aperture sizes. Finally, in the fifth section, we make an experimental demonstration of the validity of our calculations. This demonstration is carried out for two different beams at two different wavelengths (1030 nm and 1550 nm).

We consider that the presented method can be of general application, helping for reliable comparisons and thus in solving some persistent discrepancies on fluence threshold determination that are due to methodology issues. Additionally, the advent of new laser sources in different parts of the spectrum, and in particular in the infrared domain that hold promises for new scientific and industrial applications, supports the timeliness of this report to set a general criterion for accurate determination of the fluence and that is not dependent to a metrology technology.

## 2. Liu’s method

Liu’s method (or D-square method) refers to a simple experimental approach that allows determination of the fluence ablation threshold by measurements of the sizes of induced modifications at different irradiation energies ^19^, without the need for imaging the beam profile. This methodology assumes a Gaussian laser beam profile, being expressed mathematically as,

F(r)=F0⋅e−2r2w02,Eq.1

where *F(r)* is the local fluence at a given radial position, *r*, *F*_0_ the peak fluence value and *w*_0_ the radial Gaussian beam waist at 1/ *e* ^2^ the peak value. Liu’s method, under the hypothesis of a deterministic ablation behaviour, defines the ablation fluence threshold, *F _th_*, as the local fluence corresponding to the border of the crater, exhibiting a radius equal to *R*. Therefore, the following expression is obtained,

Fth=F0⋅e−2R2w02,Eq.2

expression that can be transformed into a linear relationship by taking its logarithm, leading to:

$$\;R^{2}=\frac{w_{0}^{2}}{2}\ln\;\left(\frac{F_{0}}{F_{th}}\right)\;.\;\text{Eq.}\;3$$

However, the experimental parameter that is usually measured is not the peak fluence but the integrated pulse energy, *E*. The relationship between those parameters is linear, related with effective beam area ( *EBA*). This is a general relationship (independent of the beam shape) and is obtained by the 2D-integration of the fluence distribution ^27^,

$$E=\iint_{S}F(x,y)\;ds=F_{0}\cdot EBA\;\text{Eq.}\;4$$

Calculating this integral for the Gaussian function ( Equation 1) in polar coordinates ( *dS* = *r dr dθ*), one obtains $EBA=\pi w_{0}^{2}/2,$ leading to the well-known relationship:

$$\;F_{0}=\frac{2E}{\pi w_{0}^{2}}\;\text{Eq.}\;5$$

Accordingly, by defining the energy ablation threshold, *E _th_* as the minimum pulse energy at which ablation is produced, the fluence ablation threshold is calculated as,

$$\;F_{th}=\frac{2E_{th}}{\pi w_{0}^{2}}\;\text{Eq.}\;6$$

By substituting Equation 5 and Equation 6 in Equation 3, we establish the relationship between the observables *R* (or the ablated area, *A* = *πR* ^2^) and *E*,

$$\;R^{2}=\frac{w_{0}^{2}}{2}\;\ln\left(\frac{E}{E_{th}}\right)\;\text{Eq.}\;7$$

This relationship is the key point of the Liu’s method. The latter proposes to represent on the x-axis the “ln( *E*)” values and on the y-axis the “ *R ^2^*” values. Therefore, the Gaussian beam waist ( *w _0_*) is retrieved through the slope of a linear regression. Additionally, even if it was not mentioned on the original paper of Liu, throughout the x-axis intercept the energy threshold value ( *E _th_*) can be retrieved. Then, the laser fluence threshold value, *F _th_*, can be simply calculated by applying Equation 6 with the two retrieved values.

This method is also called as the D-square-method according to the representation of graphics with diameter values ( *D* ^2^) on the y-axis. Then, the Equation 7 turns into: $D^{2}=2w_{0}^{2}\;\ln(E/E_{th})$ .

## 3. Extension of Liu’s method for an Airy Disk: mathematical description and correction factors

An Airy disk is the diffraction pattern obtained at the focal position resulting from a uniformly illuminated circular aperture. It is described mathematically in polar coordinates as,

$$F(r)=F_{0}{\left[\frac{2\cdot J_{1}(r^{\prime })}{r^{\prime }}\right]}^{2}\;\text{with}\;r^{\prime }=\frac{r\cdot 2.5838}{w_{Airy}}.\;\text{Eq.}\;8$$

*J*_1_( *r′*) is the Bessel function of the first kind of order one and *w _Airy_* the radial waist at 1/ *e* ^2^ the peak value introduced with a change of variable. As an equivalence from the previous section for a Gaussian beam, the fluence ablation threshold is described as,

$$F_{th}=F_{0}{\left[\frac{2\cdot J_{1}(R^{\prime })}{R^{\prime }}\right]}^{2}\;\text{with}\;R^{\prime }=\frac{R\cdot 2.5838}{w_{Airy}},\;\text{Eq.}\;9$$

with *R* the radius of the crater.

**Figure 1.**
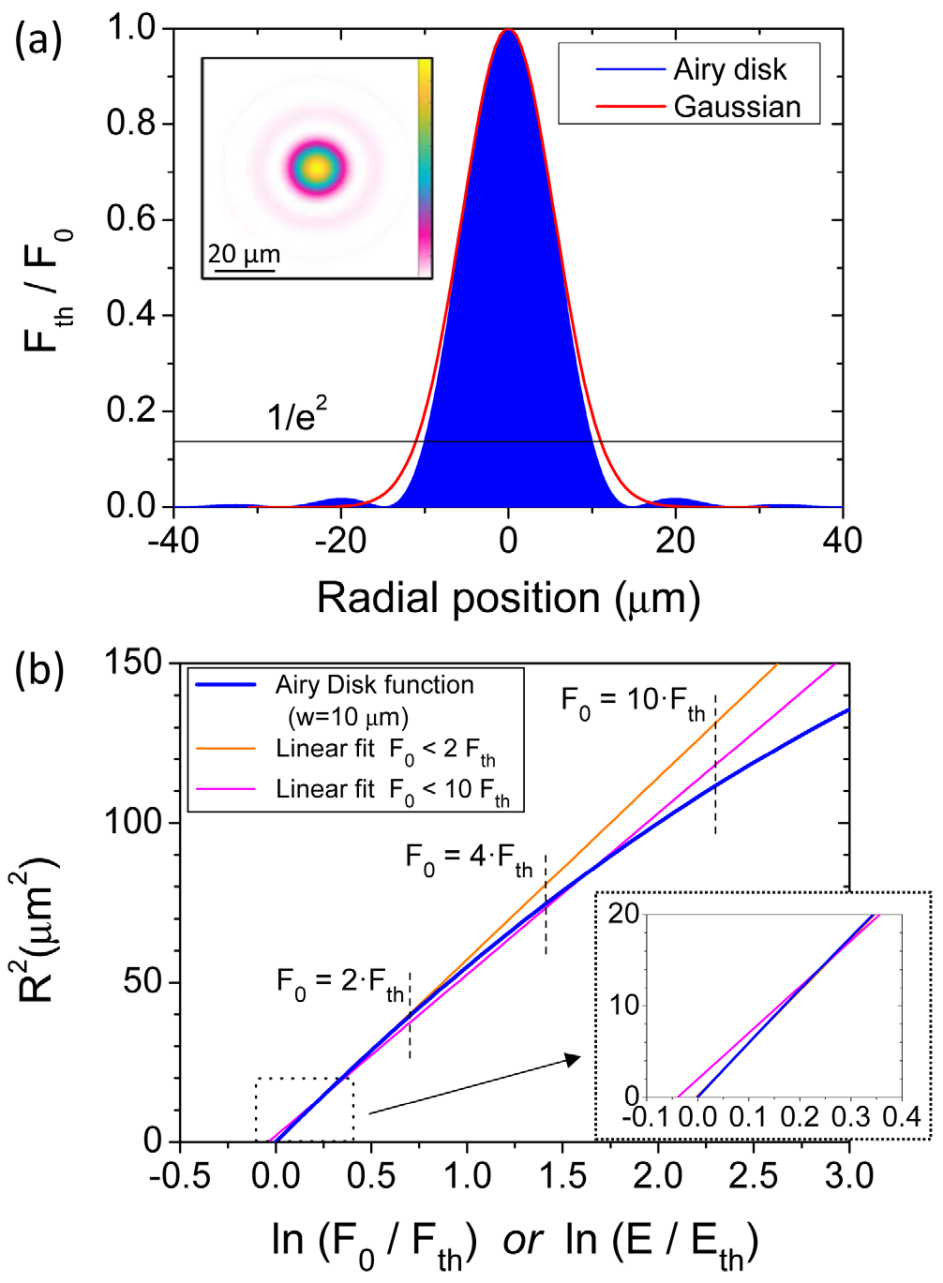
( **a**) Numerically calculated radial profile of an Airy disk ( *w _Airy_* = 10 *μm*) and a Gaussian beam both having an identical effective beam area (EBA. The inset is the 2D profile of the Airy disk. ( **b**) Representation according to Liu’s method for the Airy disk represented in ( **a**) and two linear regressions for different ranges of considered fluences: one with fluences up to 2 times above the ablation threshold (2 ⋅ *F _th_*) and another up to 10 times (10 ⋅ *F _th_*). ( *F*_0_ is the pulse peak fluence, *F _th_* is the fluence threshold for ablation, *E* is the pulse energy, *E _th_* is the energy threshold for ablation and *R ^2^* is the radius square of the induced crater).

An obvious difference with the Airy spot is in the absence of a linear relationship as the one facilitating the analyses for rigorous Gaussian beams. However, we would like to highlight here the similarities and quantify the differences between these two radially symmetric profiles. As an example, an Airy disk with a waist of *w _Airy_* = 10 *μm* is shown in Figure 1 (a). Together with this function is also represented a Gaussian beam of identical integrated pulse energy. The most important differences are visible near the pedestal of the distributions with a progressively vanishing Airy function oscillating around zero. Unfortunately, an Airy disk beam has not a simple analytical expression establishing the relationship between fluence and energy, as there is for Gaussians beams ( Equation 5). This imposes to use directly the integration formula expressed on Equation 4 for the Airy disk function ( Equation 8), which allows the numerical calculation of the corresponding *EBA*. For our represented case ( *w _Airy_* = 10 *μm*), the effective beam area value obtained is *EBA* = 188.2 *μm ^2^*. 

In order to evaluate the deviations of the Gaussian-based Liu’s method when applied for an Airy disk, we present in Figure 1(b) the relationship between *R* ^2^ and ln( *F*_0_/ *F _th_*) for our Airy case. The values are retrieved from the data shown in Figure 1 (a), doing the pertinent calculations and swapping the axes. It can be observed with Figure 1(b) a deviation from a linear behavior at relatively high excitation levels, similar to the one experimentally observed by Bonse *et al*. ^24^. Despite this trend, equivalent Gaussian functions (the closest) can be obtained by linear regressions of this graph, that is literally applying the Liu’s method. Due to the non-linear character of this curve, the result depends on the range of considered energies. As examples, in Figure 1(b) two different linear regressions are presented, one with a maximum considered energy *E* = 2 ⋅ *E _th_* and another of *E* = 10 ⋅ *E _th_*.

In Table 1 we provide the obtained parameters ( *w _0,Liu_* and *E _th,Liu_*) applying the same procedure under different maximum considered energies. The obtained equivalent Gaussian beam waists ( *w _0,Liu_*), allows for the calculation of their corresponding *EBA* $(EBA=\pi w_{0,Liu}^{2}/2).$ After comparing those values with the *EBA* obtained after the Airy disk integration a correction factor, *η _EBA_*, is obtained. Knowing the relationship between *EBA* and the peak fluence ( Equation 4) a fluence correction factor is calculated as *η _F_* = 1/ *η _EBA_*. Therefore, the peak fluence of an Airy disk for a given measured energy ( *E*) can be expressed as,

$$\;F_{0}=\frac{2\cdot E}{\pi w_{0,Liu}^{2}}\cdot \eta_{F},\;\text{Eq.}\;10$$

**Table 1.** Retrieved equivalent Gaussian waist ( *w*_0, *Liu*_) and energy threshold value ( *E _th,Liu_*) after applying Liu’s method for the Airy disk represented in Figure 1. The Liu’s method is applied considering 4 different cases with energies above the ablation threshold ( *E _th_*). The integration correction factor, *η _EBA_*, and the ablation energy threshold correction factor, $\eta_{E_{th}}$ , are obtained after comparison of the Airy disk function and the retrieved Gaussians functions using the Liu’s method. *η _F_* is the fluence correction factor.

Maximum considered energy	*w*_0, *Liu*_ (μm)	*E _th,Liu_*	*η _EBA_*	$\eta_{F}\;=\;\frac{1}{\eta_{EBA}}$	$\eta_{E_{th}}$
2·Eth	10.63	0.99	1.06	0.94	1.01
3·Eth	10.44	0.98	1.10	0.91	1.02
5·Eth	10.20	0.96	1.15	0.87	1.04
10·Eth	9.87	0.92	1.23	0.81	1.09

This expression is directly equivalent to the Equation 5 but introducing the fluence correction factor, *η _F_*, a factor that depends on the range of energy considered when the Liu’s method is applied.

Additionally, as observed with the inset of Figure 1(b), the linear fit applied to the Airy function can lead to errors on the reading of the real energy threshold by the x-intercept. For all discussed cases, this error is numerically evaluated introducing the correction factor of the ablation energy threshold, $\eta_{E_{th}}$ . Overall, the exact ablation fluence threshold when applying the Liu’s method for an Airy beam is obtained as,

$$\;F_{th}=\frac{2\cdot E_{th,Liu}}{\pi w_{0,Liu}^{2}}\cdot \eta_{E_{th}}\cdot \eta_{F}\;\text{Eq.}\;11$$

Complementing some correction factors already given in Table 1, more cases are calculated and a corresponding abacus is presented together with measurements in the following section (see in particular Figure 3).

## 4. Extension of Liu’s method for Gaussian beams truncated using circular apertures

As previously mentioned, an Airy disk is the diffraction pattern obtained at the focal position of a lens of limited aperture when irradiated by a uniform plane wave. In practice, this corresponds to a perfectly top-hat beam facing the lens, or the use of a circular aperture much smaller than the size of a nearly-Gaussian beam. Therefore, in this section we show the calculations and corrections factors to account in the Liu’s method for more realistic cases using truncated Gaussian beams with circular apertures at focusing lens position.

For obtaining the diffraction pattern for different truncation conditions we rely on the PSFLab software ^28^ for calculations based on rigorous vectorial theory. We calculate the point spread function and in particular the fluence radial profile at the focus for different focusing and illumination conditions.

The calculations account for a Gaussian profile filling parameter, *β _G_*, which is defined as the ratio between the radius of the circular aperture ( *a*) and the collimated Gaussian beam radius ( *w*): *β _G_* = *a*/ *w*. This parameter describe also the power transfer (or aperture transmission) *P*_*T*_, as 29,

$$\;P_{T}=1-\exp(-2\beta_{G}^{2})\;\text{Eq.}\;12$$

Interestingly, this power transfer is a parameter that can be easily determined in an experiment by simply measuring the laser power with and without the chosen aperture.

**Figure 2.**
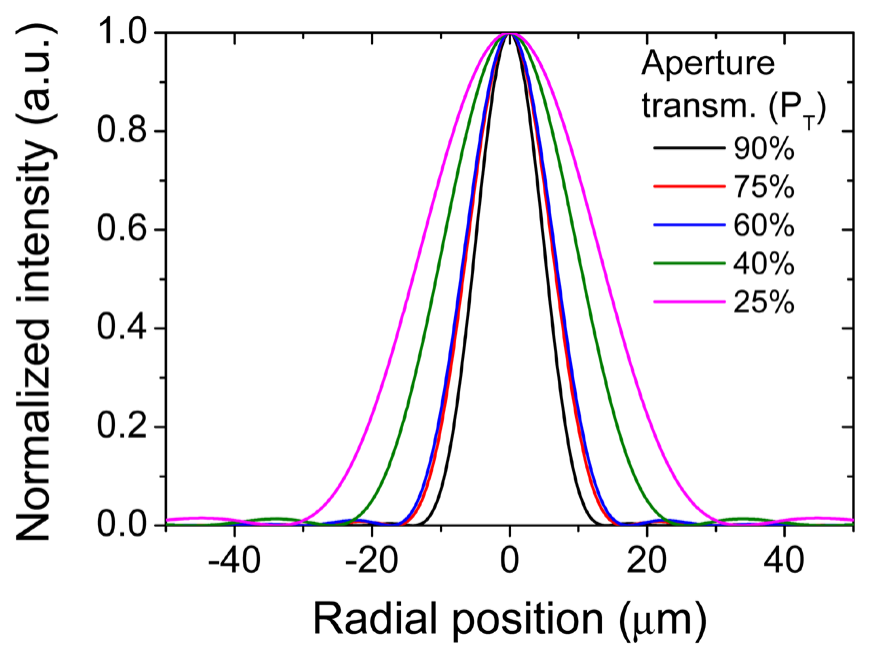
Numerically calculated radial beam profiles at focus position of a lens illuminated by a Gaussian beam truncated by circular apertures. The calculations assume the following parameters: λ=1030 nm, focal lens of 50-mm, an incident collimated Gaussian beam diameter of 5-mm ( *2·w*) and varying aperture diameter adapted to correspond to the mentioned aperture transmissions. Data are obtained by using PSFLab software ^28^.

In Figure 2, we show the obtained profiles for different power transfer values, ranging from 90 % to 25 %. In this particular case, the parameters used for the calculations were: λ=1030 nm, focal lens of 50-mm, an incident collimated Gaussian beam with diameter of 5-mm ( *2·w*) and aperture diameter values ( *2·a*) adapted to fit the chosen aperture power transfer ( Equation 12).

All profiles in Figure 2 exhibit a ring structure, even if hardly visible with a linear scale in intensity. The relative intensities of the rings are represented in Table 2 and compared with those of a perfect Airy disk ( *β _G_* = 0). Additionally, the energy distribution on the central region and the rings is also represented on Table 2, after a numerical calculation of the *EBA* for the full function ( Equation 4) and the contribution of each part of the ring structure. The table provides already useful information on the characteristics of the ring functions resulting when truncating a Gaussian beam with circular apertures. Even if performed for a specific wavelength and focusing conditions, it is important to highlight the generality of these calculations that only depend on the filling parameter *β _G_*.

**Table 2.** Maximum intensity and energy distribution analysis of the different parts of the resulting beam profiles from truncated Gaussian beams (circular aperture) shown in Figure 1 (a) and Figure 2.

Ring function	Peak intensity (a.u.)			% Energy distribution			
	Central region	First ring	Second ring	Central region	First ring	Second ring	Rest
Airy ( *β _G_* ≈ 0)	1	1.8·10 ^-2^	4.2·10 ^-3^	83.8 %	7.2 %	2.8 %	6.2 %
P _T_ =25% ( *β _G_* =0.38)	1	1.5·10 ^-2^	3.6·10 ^-3^	86.4 %	6.2 %	2.4 %	5.0 %
P _T_ =40% ( *β _G_* =0.51)	1	1.3·10 ^-2^	3.2·10 ^-3^	87.9 %	5.5 %	2.1 %	4.5 %
P _T_ =60% ( *β _G_* =0.68)	1	1.1·10 ^-2^	2.6·10 ^-3^	90.5 %	4.2 %	1.7 %	3.6 %
P _T_ =75% ( *β _G_* =0.83)	1	7.7·10 ^-3^	1.9·10 ^-3^	93.0 %	3.0 %	1.2 %	2.8 %
P _T_ =90% ( *β _G_* =1.07)	1	3.7·10 ^-3^	1.1·10 ^-3^	96.5 %	1.3 %	0.7 %	1.5 %

**Figure 3.**
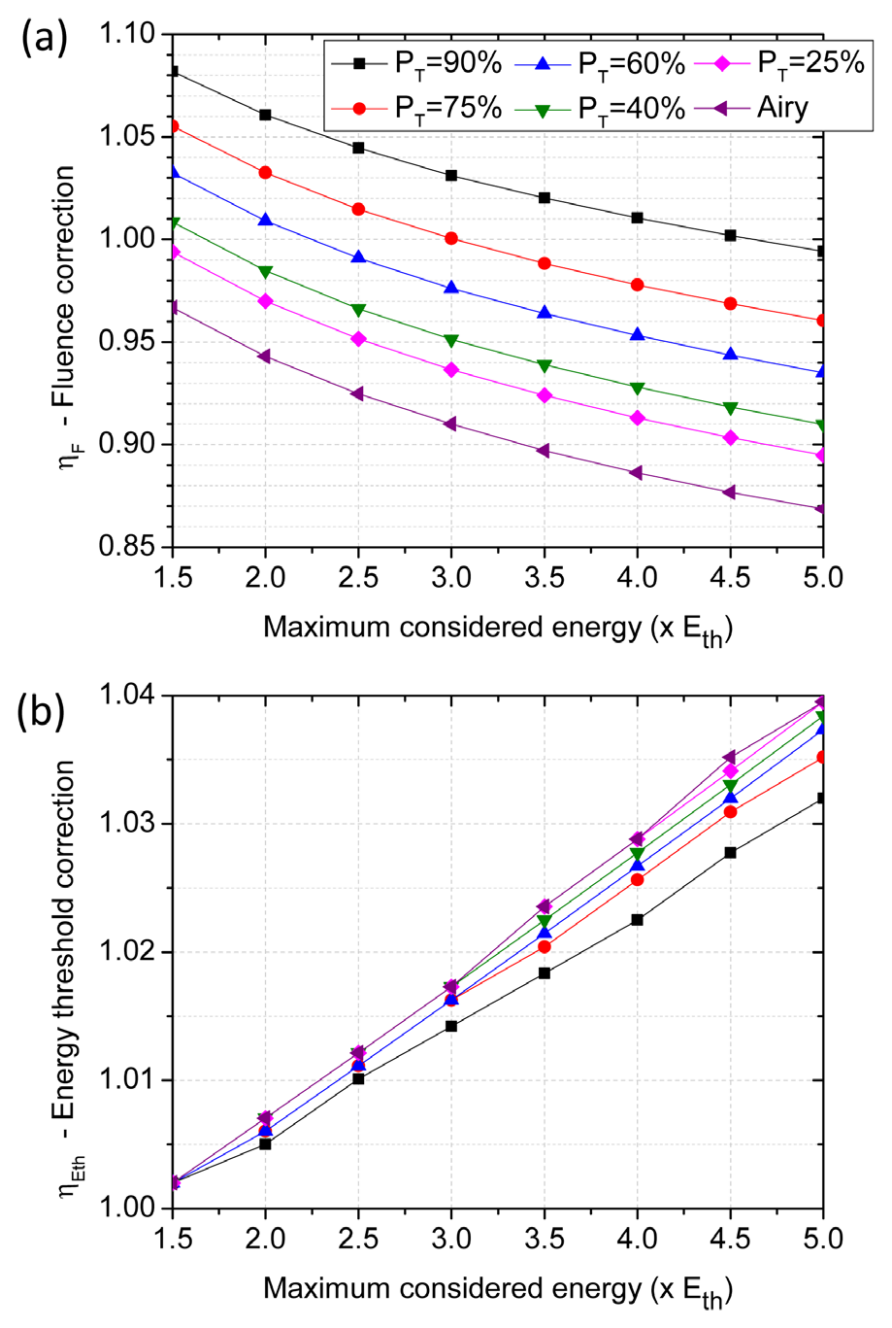
( **a**) Correction factor, *η _F_*, to apply to the fluence calculation ( Equation 10) when irradiating with truncated beams by a circular aperture. ( **b**) Correction factor, *η _E _th__*, to apply to the fluence threshold calculation ( Equation 11) when irradiating with truncated beam by a circular aperture. In both cases, the horizontal axis corresponds to the maximum considered energy for the linear regression of the Liu’s method. Straight lines joining the points serve for view guiding.

With the obtained beam profiles, a representation similar to the one plotted in Figure 1 (b) is performed for each function (not shown). Applying Liu’s method representation and the same linear regression analyses described on the previous section, correction factors are obtained. Again, the correction factor depends on the range of considered energies for the linear regression. The results of these calculations are represented in Figure 3 for various energy ranges, with energy maxima up to five times the energy threshold (5 ⋅ *E _th_*). 

We observe from Figure 3(a) that the introduction of a fluence corrective factor is needed even if the beam spot is the result of a moderate truncation ( *e.g.* *P _T_* = 90%). This is important since one may intuitively expect negligible consequence of a moderate truncation for the validity of a Gaussian approximation. By helping in symmetrizing an imperfect incoming Gaussian beam one may have considered the truncation of beam as an improvement to approach the Gaussian profile assumption needed to apply Liu’s method. While this filtering may be beneficial for the validity of Liu’s method representation, our analysis indicate one should not ignore that the resulting peak fluence and fluence threshold values will remain biased unless the appropriate correction factors, as those calculated in Figure 3, are applied in Equation 10 and Equation 11.

For the practical case of Gaussian beams truncated by using circular apertures, another interesting conclusion from Figure 3 (b) is that the correction factor of the energy threshold, $\eta_{E_{th}}$ is less significant than the correction associated to the fluence distribution, *η _F_*. In order to make data more accessible for potential users of this methodology, we present the correction factors, *η _F_* and *η _E _th__* in Table 3 and Table 4.

**Table 3.** Correction factor, *η _F_*, to apply to the fluence calculation ( Equation 10) when irradiating with truncated beam by a circular aperture. The values are represented graphically on Figure 3(a).

*η _F_*		Aperture power transfer ( *P _T_*)				
		90%	75%	60%	40%	25%
**Maximum considered energy**	**1.5·Eth**	1.082	1.055	1.032	1.008	0.994
	**2·Eth**	1.061	1.033	1.009	0.985	0.970
	**2.5·Eth**	1.045	1.015	0.991	0.966	0.952
	**3·Eth**	1.031	1.000	0.976	0.951	0.936
	**3.5·Eth**	1.020	0.988	0.964	0.939	0.924
	**4·Eth**	1.010	0.978	0.953	0.928	0.913
	**4.5·Eth**	1.002	0.969	0.944	0.918	0.903
	**5·Eth**	0.994	0.960	0.935	0.910	0.895

**Table 4.** Correction factor, *n _E _th__*, to apply to the fluence threshold calculation ( Equation 11) when irradiating with truncated beam by a circular aperture. The values are represented graphically on Figure 3(b).

*η _E _th__*		Aperture power transfer ( *P _T_*)				
		90%	75%	60%	40%	25%
**Maximum considered energy**	**1.5·Eth**	1.002	1.002	1.002	1.002	1.002
	**2·Eth**	1.005	1.006	1.006	1.007	1.007
	**2.5·Eth**	1.010	1.011	1.011	1.012	1.012
	**3·Eth**	1.014	1.016	1.016	1.017	1.017
	**3.5·Eth**	1.018	1.020	1.021	1.022	1.024
	**4·Eth**	1.022	1.026	1.027	1.028	1.029
	**4.5·Eth**	1.028	1.031	1.032	1.033	1.034
	**5·Eth**	1.032	1.035	1.037	1.038	1.040

## 5. Experimental validation and practical relevance

### A. Experimental configuration

In this section, we show two experiments validating the use of the proposed corrected extension of the Liu’s method for a beam truncated with a circular aperture. We show the superior relevance and robustness for threshold determinations for beams deviating from a perfect Gaussian profile. This is because the truncation tends to create, independently of incoming beam, a more controlled Airy-like pattern for which rigorous correction factors can be derived for the application of Liu’s method (originally proposed for Gaussian beam only).

In the first case, this methodology was applied for a beam at 1030-nm wavelength directly generated by a femtosecond laser amplifier (Pharos, Light Conversion). On the second case, it was applied for a beam at 1550-nm wavelength generated through non-linear processes on an optical parametric amplifier (OPA, Orpheus-HP, Light Conversion). The pulse durations at both laser wavelengths were characterized by an autocorrelator (TiPA, Light Conversion), being of 170 fs at 1030 nm and 190 fs at 1550 nm.

The irradiation set-up was composed of a variable circular aperture (SM1D12C, Thorlabs) positioned as close as possible to an, aspheric lens of f=50 mm (117-2550, Eskma) and a XYZ-motorized sample holder. Single-shot irradiations were controlled by a pulse-picker integrated in the laser system. Pulse energies were externally adjusted by a set of broadband metallic filters. The irradiated sample of reference was a sapphire window of 1 mm thickness and c-cut orientation. The choice of this target material was motivated by the very neat craters produced with ultrashort pulses without apparent thermally affected zones ^13^, being consequently considered as a reference dielectric for impact-based beam characterization methods. Additionally, a fused silica sample (UV-fused silica) was also used on the experiments, given the fact that it is probably the most studied dielectric material. The numerous damage fluence thresholds values reported in the literature are important for comparisons and validation of the methodology of this article. In all cases, our damage criterion is ablation, which is determined by measuring the profiles of irradiated areas by a confocal microscope (Leica DCM3D, 460 nm illumination, 150× objective lens). Examples of the images obtained under this microscope can be found in our previous publications ^13, 30^.

Additionally, an imaging system composed of a microscope objective (Mitutoyo 100X NA-0.5, or Mitutoyo 50X of NA-0.42), a tube lens and infrared camera (Raptor OWL 640), mounted in a micrometric XYZ stage, was used to obtain the beam image at the focal position. In order not to introduce any distortion on the beam, images are recorded at low energies. For each image a background subtraction procedure is applied on the basis of another reference image captured after blocking the laser beam. The 16-bit intensity image is normalized after dividing by the peak intensity value in the measured distribution. To account for the magnification of the imaging system, the image is re-scaled after imaging a resolution test target. With the image resulting from those operations the *EBA* in *μm* ^2^ is obtained by numerical 2D-integration.

Data produced for validation are available from Zenodo as underlying data ^31^.

### B. Ablation test experiment at 1030-nm

**Figure 4.**
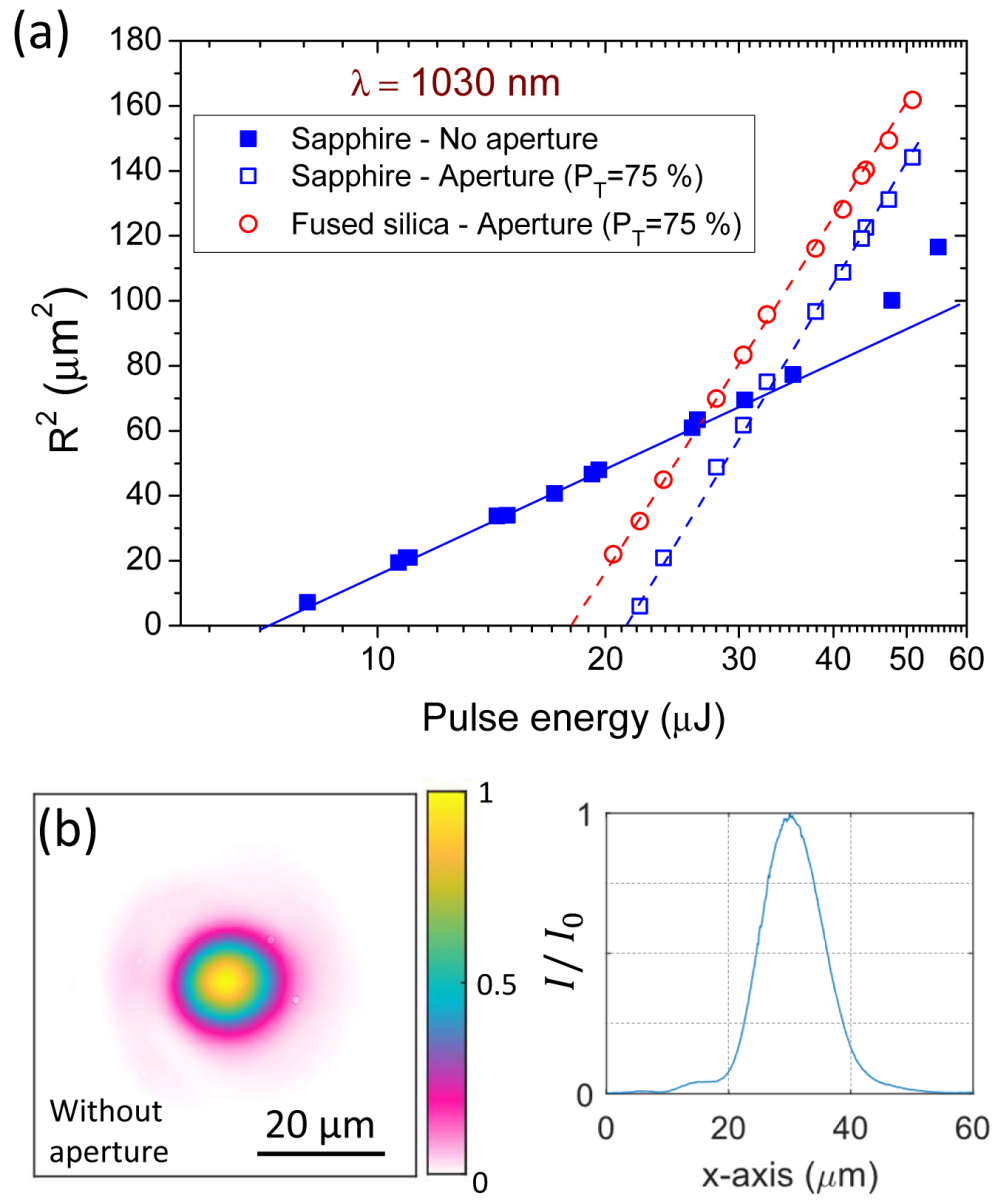
( **a**) Ablated areas in sapphire and fused silica as a function of the pulse energy. Craters are produced by single pulse irradiation (170-fs) at 1030 nm with two different beam profiles depending on the presence or not of a circular aperture ( *P _T_* = 75 %). ( **b**) (left) Image of the beam without any aperture at the best focal position (f=50 mm) as captured by an imaging system equipped with InGaAs array detector. (right) Horizontal beam profile at the central position of the beam image.

The results of the modifications induced in sapphire and fused silica with pulses of 170-fs at 1030-nm wavelength are shown on Figure 4 (a). The representation follows the Liu’s method (see Equation 7) with an x-axis for the energy in logarithmic scale to perform linear regressions on the data. In particular, two experiments were performed on the sapphire sample. The first one is for a beam directly focused on the target surface and second one is for the same configuration but placing a circular aperture before the lens adjusted for a power transfer of 75%.

The crater measurements without the pinhole are represented with solid squares. A nearly perfect linear behavior is observed up to energies of about 40 µJ, which corresponds to excitation levels of ∼6 times the ablation energy threshold. This deviation could be associated to air ionization affecting the propagation of intense beams ^32^. However, the corresponding irradiation intensity in our experiment (≈ 10 ^14^ W/cm ^-2^) does not directly support this hypothesis. The deviation is more surely explained through the analysis of the beam image at the focal position shown in Figure 4 (b). On this beam image and its horizontal cross-section, we observe a pedestal surrounding an almost perfect Gaussian profile. The influence of this pedestal becomes visible on the modifications only when irradiating well-above the ablation fluence threshold, as observed on Figure 4 (a). However, more importantly, this leads to a significantly biased fluence threshold determined by the original Liu’s method due to some energy distributed outside the assumed Gaussian beam profile, as explained in the following paragraph.

When applying the Liu’s method taking all the values below 40 µJ, we obtain *E _th,Liu_* = 7.1 μJ and *ω _Liu_* = 9.7 μm. The corresponding full-width at half maximum is *FWHM _Liu_* = 11.4 μm, being in excellent agreement with the value obtained by imaging as *FWHM _Image_* = 11.6 μm. Applying Equation 6, this leads to the fluence threshold determination *F _th,Liu_* = 4.8 J/cm ^2^. Alternatively, an integration of the fluence distribution can be obtained with the image shown in Figure 4 (b), leading to a value of *EBA* = 165 *μm* ^2^. By using the ablation energy threshold obtained with the Liu’s method and applying the relationship described in Equation 4, we obtain for a rigorous determination of the threshold: *F _th,Image_* = 4.3 J/cm ^2^, showing a 11.6% difference when comparing with the value obtained with the Liu’s method. This difference directly comes from the perfect Gaussian beam assumption used in Liu’s method. Its validity is not strictly fulfilled in the considered case and we dare say in most of experiments as real beams always exhibit more or less imperfections.

According to the craters produced in sapphire when placing a circular aperture setting a power transfer of *P _T_* = 75%, the linear regression corresponding to Liu’s method, also shown in Figure 4(a), leads to *E _th,Liu_* = 21.1 μJ and *ω _Liu_* = 18.1 *μm*. The maximum considered energy for the fit equals to 50.9 μJ, corresponding to 2.41 ⋅ *E _th_*. Therefore, under this experimental conditions and looking at Figure 3 (or Table 3 and Table 4), we extract the following correction factors: *η _F_* = 1.02 and $\eta_{E_{th}}$ = 1.01. Applying those correction factors and the regression parameters obtained by Liu’s method into Equation 11, a fluence threshold value of *F _th_* = 4.2 J/cm ^2^ was obtained, which is very close (2.3% difference) to the value obtained by complete numerical integration of the beam profile (see above).

Finally, this methodology is applied also for fused silica. The conventional Liu’s method gives *E _th,Liu_* = 17.9 μJ and *ω _Liu_* = 17.6 μm. According to the maximum considered energy for linear regression, 47.4 μJ (2.64 ⋅ *E _th_*), we extract the following correction factors: *η _F_* = 1.01 and $\eta_{E_{th}}$ = 1.01 and we finally obtain *F _th_* = 3.8 J/cm ^2^. Validation of this result by comparing with values in the literature is not simple, since the reported values exhibit large differences, as summarized by Gallais *et al.* ^5^ when comparing the fluence threshold values obtained by different authors in fused silica under irradiations at 800 nm or 1053 nm. Even though comparisons are difficult, we observe that our value is in good agreement with the value of 3.9 J/cm ^2^ obtained by Winkler *et al.* ^33^, performed with pulses with characteristics close to the ones used in our experiment (200 fs pulse duration at 800 nm).

We can conclude, after demonstrating the obtention of similar values by two different means (beam imaging and our presented methodology), that controlled truncation of the beam leads to an improved reliability and confidence on the fluence threshold metrology. This experimental approach can be useful to solve some of the discrepancies observed in the literature due to works relying generally on Liu’s method without detailed analyses on the real beam profile.

### C. Ablation test experiment at 1550 nm

**Figure 5.**
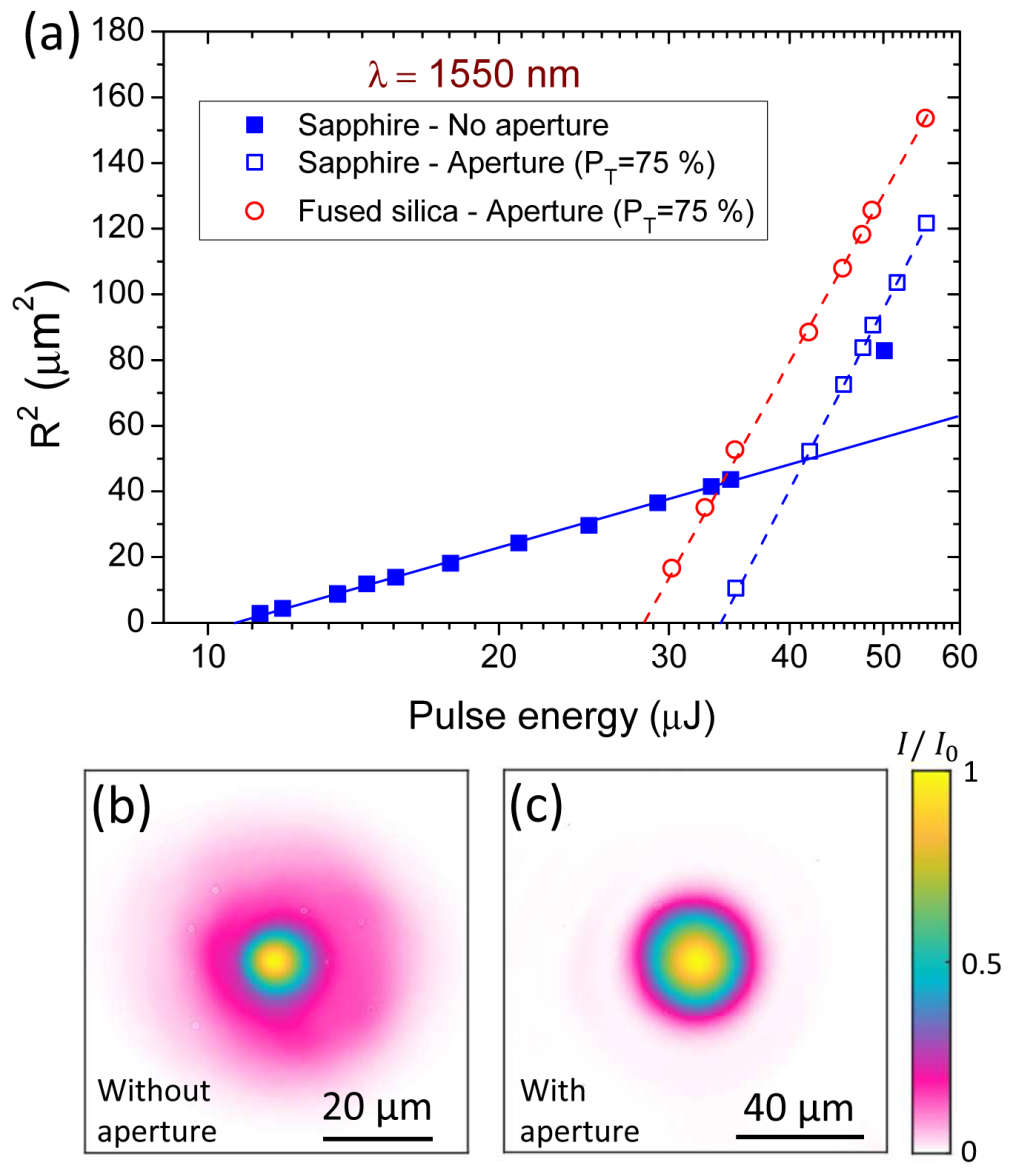
( **a**) Ablated areas in sapphire and fused silica as a function of the pulse energy. Craters are produced by single pulse irradiation (190-fs) at 1550 nm with two different beam profiles depending on the presence or not of a circular aperture. ( **b**) Beam image produced at the focal position after directly focusing the OPA beam. ( **c**) Beam image produced at the focal position after focusing the same beam but inserting a circular aperture ( *P _T_* = 75%) before the lens.

To further illustrate the benefit from the proposed simple method, we show in Figure 5 (a) the results of an ablation experiment by irradiation with single 190-fs pulses at 1550-nm wavelength. We compare the measurements obtained by using directly the beam delivered by the OPA (without any aperture) to those obtained with a truncated beam using an aperture set again for a power transfer of 75%. Before discussing on the results, it is worthy to look at Figure 5 (b–c), where the corresponding beam profiles at the focal position are represented. In Figure 5 (b), the beam image produced after focusing the OPA beam (without aperture) shows a notorious deviation from a perfect Gaussian beam. This beam shape, with the presence of a large pedestal where an important part of the energy is present, clearly makes Liu’s method not applicable. This point will be confirmed just after.

Moreover, this imperfect shape at the focal position also suggests that the beam before the lens is also imperfect and not Gaussian. Accordingly, the Fourier transformed function resulting from the application of a circular aperture is not necessarily corresponding to the rigorous analysis made in section 4, unless the aperture is sufficiently closed. Figure 5 (c) shows the beam profile resulting after placing a circular aperture with *P _T_* = 75%. A central spot and a surrounding ring are observed, where the maximum value of the ring (after a circular analysis with ImageJ software) corresponds to 7.4 ⋅ 10 ^-3^. This is very close to the theoretical value of 7.7·10 ^-3^ in Table 2 and confirms the applicability of the analysis made in section 4 suggesting that the cut pedestal was constituting the main difference with a Gaussian beam. In view of this case, it is interesting to retain *P _T_* = 75% as an appropriate level of truncation but even stronger truncation may have been needed depending on the beam quality.

We now return to the analysis of the results shown on the Figure 5 (a). When applying the Liu’s method to the values obtained without an aperture we obtain an ablation threshold value for sapphire of 7.8 J/cm ^2^. Due to the presence of a considerable energy in the pedestal of the beam profile and ignored by the method, this can be considered as an incorrect value. Applying the same methodology for the data obtained by placing the circular aperture, *E _th,Liu_* = 33.9 μJ and *ω _Liu_* = 22.1 μm is obtained. According to the range of considered energies for linear regression, up to 55.4 μJ (1.63 ⋅ *E _th_*) the correction factors to introduce in Equation 11 are : *η _F_* = 1.05 and $\eta_{E_{th}}$ = 1.00. Finally we obtain *F _th_* = 4.6 J/cm ^2^. This illustrates the enormous fluence determination error (>60 %) when directly applying the Liu’s method for our OPA imperfect beam. Performing the same procedure for fused silica, we obtained: *E _th,Liu_* = 28.1 μJ, *ω _Liu_* = 22.3 μm, *E _max_* = 2.0 ⋅ *E _th_*, *η _F_* = 1.03, $\eta_{E_{th}}$ = 1.01 and finally *F _th_* = 4.1 J/cm ^2^.

The values calculated by applying Equation 4 after numerical integration of the beam image ( Figure 5(c)), giving a *EBA* = 680 μm ^2^, are *F _th_* = 5.0 J/cm ^2^ for sapphire and *F _th_* = 4.2 J/cm ^2^ for fused silica, overestimating the values obtained by the proposed method of this article. This observation highlights the main limitation of fluence threshold determination accuracy when using beam imaging, since its precision lies on a perfect spatial calibration and on rigorous integration of the entire beam details. The latter consideration corresponds to ideal imaging conditions that are not accessible experimentally. In our particular case, the slightly overestimated threshold values can be associated to an underestimation of the EBA, since the intensity of secondary diffractive rings do not overpass the noise level of the IR-camera used for imaging. Considering this aspect, our obtained threshold values becomes very consistent and supports the appropriateness of the proposed analysis based on Liu’s method by introducing correction factors.

### D. Relevance, precision and recommendations

The proposed method of general application shows the ability to improve the reliability of the fluence threshold determination without the need for rigorous beam profile analyses. In this context, we anticipate its usability for fluence threshold determination at non-conventional wavelengths, in which beam imaging can become complex or, for the most extreme cases, impossible with the available sensor technologies. In particular and after the demonstration at 1550-nm, we consider that this methodology will be really useful for the fluence threshold determination on the short-wave (SWIR) and mid-wave infrared (MWIR) ranges. Those spectral ranges are of increasing interest for the laser material processing community due to the development of new sources ^34, 35^. However, there are challenges remaining for high quality beams with these new developments. This must make very appropriate the application of the method presented in this article.

Additionally, this method is not only useful for fluence threshold determination but also for peak fluence determination of an unknown beam. For doing that, after obtaining the fluence threshold with a truncated beam by a circular aperture, the peak fluence of an unknown beam (for example the original untruncated beam) is calculated following this expression,

$$\;F_{0}=F_{th}\cdot \frac{E}{E_{th}}\;\text{Eq.}\;13$$

Where *F*_0_, *E* and *E _th_* are respectively the peak fluence, the pulse energy and the energy threshold of modification of the unknown beam, and *F _th_* is the fluence threshold value obtained after irradiating with a truncated beam using a circular aperture. *E _th_* would be the only parameter to be obtained by irradiating with the unknown beam.

Although the method presented has been shown to be efficient for accessing a robust metrology in threshold determination, some aspects should be commented for a proper usability and accuracy quantification:

1. The presented correction factors are only valid if the sample is situated on the focal position or close to it (Rayleigh length). Otherwise, the beam spot will not show an Airy-like beam shape and the described analysis is not applicable.
2. It remains crucial for accurate fluence threshold determination to have sufficient data at near threshold conditions (<2 ⋅ *E _th_*), as on the classical Liu’s method. Otherwise, it constitutes a new source of error, both on the *E _th_* and *w _0_* determination, that is not considered in our report.
3. The energy of irradiation in the experiments should remain under the condition for air ionization and nonlinear propagation effects (e.g. defocusing) affecting the spatial characteristics of the delivered beam ^12^. This is of especial relevance when irradiating materials exhibiting relatively high fluence thresholds ( *e.g.* dielectrics). It is mainly for these practical considerations that we have limited our calculations of the correction factors to levels below 5 *E _th_* ( Figure 3).
4. It is estimated, if the other considerations are fulfilled, that the obtained fluence value should not be expressed with a better accuracy of ±5 %. This percentage is a conservative estimation accounting for the following aspects: possible uncertainties on selecting the most appropriate correction factors (especially when extrapolations of theoretical calculations of Figure 3 are needed to describe the practical case) or particular material responses interfering on the measured ablated area (as for example the melted rims formed in some glasses, ^9, 13^). On this second aspect, imprecisions due to subjective ablated area determination are not considered here, since our metrology is based on user-independent analysis as referred on a previous publication ^30^.

Finally, as recommendations for the level of truncation to be applied, we have observed that *P _T_* = 75% is an appropriate level when having a pseudo-Gaussian beam with a pedestal ( section 5B and section 5C). In the case of more irregular beams or totally unknown beams, we propose to perform experiments under two levels of truncation, one with moderate truncation ( *P _T_* ≥ 75%) and another with strong truncation ( *P _T_* ≤ 60%). Obtaining similar fluence values under the two experiments would indicate that a moderate truncation level is sufficient to transform the beam into an Airy-like beam. Otherwise, the most accurate fluence threshold value is obtained under the strongest truncation, since strong spatial filtering reduces the influence of irregularities of the input beam.

## 6. Conclusions

In the present work, we have explored the validity limits of the Liu’s method ^19^ which is widely applied for its usefulness for rapid assessment of material modification thresholds and achievable resolutions. The method has two requirements: (i) a strict threshold response of the material without surrounding affected zones and (ii) a perfectly Gaussian beam profile impinging on the target. While we have investigated the first of these requirements in recent works ^13, 30^, we have concentrated here on the more technical question of the importance of the beam profile. An important conclusion is that a modest deviation to the ideal Gaussian can lead to significant errors in threshold determinations. By calculating and measuring the errors associated with more or less diffracted imperfect beams, we show that errors exceeding 20% can be easily caused by beam imperfections that are undiagnosed if only the produced craters are analysed. This is because the upper part of most laser spot distributions can be advantageously compared to a Gaussian function, and so exhibit a linear dependence of the area to the logarithm of the energy in a more or less extended range of energies above threshold. The only strictly rigorous solution to this problem is to measure the beam for accurate determination of the profile and systematic numerical comparisons with the spatial characteristics of the produced modifications. However, such accurate measurement is not always possible (depending on radiations and associated measurement technologies) and its non-necessity represents actually the direct benefit and interest of the Liu’s method.

For this reason, another important contribution with this report is with the introduction of a simple extension of Liu’s method to solve this limitation. The quantitative determination of the needed correction from a truncated Gaussian beam (depending on the data considered) suggests that the introduction of a partially-closed aperture can always be used to produce a better-defined profile on target. While the associated Airy-disk pattern is in principle inappropriate to use the Liu’s method, we have shown it leads to a superior reliability in threshold determination provided that the correction factors derived in this report are applied for compensation.

The reported findings give a comprehensive vision of the measurement limitations that can explain some of the strong discrepancies existing in the literature reporting damage thresholds. A general problem with norms and standards existing on this question ^36^ is on the *a priori* knowledge of all experimental conditions that is not all always accessible. The general applicability of error compensation on apertured beams makes it particularly interesting because it improves the measurement reliability without any change of the currently widely used experimental methodology.

## Methods

Numerical calculation of the beams for different truncation conditions are obtained with PSFlab software ^28^ (version 3.5).

The calculation of Liu’s method parameters ( section 3, section 4 and section 5), the obtention of the Airy disk function ( section 3) and the calculation of the integrals enabling to obtain the EBA ( section 3 and section 4) are performed by programming respectively the Equation 7, Equation 8 and Equation 4. In this manuscript those calculations were performed by using MATLAB (R2020a under a licence of Universidad Autónoma de Madrid). Other programming languages (e.g. Python or C++) would be also appropriate for those purposes.

Analysis of experimental beam images is performed by using ImageJ software (version 1.53a). 

The complete experimental methodology is detailed on section 5A.

## Data availability

### Underlying data

Zenodo: Raw data for manuscript "Simple and robust method for determination of laser fluence thresholds for material modifications: an extension of Liu's approach to imperfect beams". http://doi.org/10.5281/zenodo.4421003 ^31^

This project contains the following underlying data:

- PSFlab_raw_1030nm-f50mm-T25.txt (PSFlab raw data of *P _T_* = 25% profile represented in Figure 2).
- PSFlab_raw_1030nm-f50mm-T40.txt (PSFlab raw data of *P _T_* = 40% profile represented in Figure 2).
- PSFlab_raw_1030nm-f50mm-T60.txt (PSFlab raw data of *P _T_* = 60% profile represented in Figure 2).
- PSFlab_raw_1030nm-f50mm-T75.txt (PSFlab raw data of *P _T_* = 75% profile represented in Figure 2).
- PSFlab_raw_1030nm-f50mm-T90.txt (PSFlab raw data of *P _T_* = 90% profile represented in Figure 2).
- ForLiu_log-and-R2-25T.dat (Treated data from PSFlab_raw_1030nm-f50mm-T25.txt for directly applying the Liu’s method. The first column represents the irradiation energy in log scale and the second column represents the squared crater radius).
- ForLiu_log-and-R2-40T.dat (Treated data from PSFlab_raw_1030nm-f50mm-T40.txt for directly applying the Liu’s method. The first column represents the irradiation energy in log scale and the second column represents the squared crater radius).
- ForLiu_log-and-R2-60T.dat (Treated data from PSFlab_raw_1030nm-f50mm-T60.txt for directly applying the Liu’s method. The first column represents the irradiation energy in log scale and the second column represents the squared crater radius).
- ForLiu_log-and-R2-75T.dat (Treated data from PSFlab_raw_1030nm-f50mm-T75.txt for directly applying the Liu’s method. The first column represents the irradiation energy in log scale and the second column represents the squared crater radius).
- ForLiu_log-and-R2-90T.dat (Treated data from PSFlab_raw_1030nm-f50mm-T90.txt for directly applying the Liu’s method. The first column represents the irradiation energy in log scale and the second column represents the squared crater radius).
- 1030nm-Sapphire-NoAperture-Energy_VS_R2.dat (Experimental data of squared crater radius (second column) produced with different irradiation energies (first column) in a sapphire sample. Irradiation wavelength 1030 nm. No aperture: *P _T_* = 100%).
- 1030nm-Sapphire-Aperture-Energy_VS_R2.dat (Experimental data of squared crater radius (second column) produced with different irradiation energies (first column) in a sapphire sample. Irradiation wavelength 1030 nm. Aperture: *P _T_* = 75%).
- 1030nm-FusedSilica-Aperture-Energy_VS_R2.dat (Experimental data of squared crater radius (second column) produced with different irradiation energies (first column) in a fused silica sample. Irradiation wavelength 1030 nm. Aperture: *P _T_* = 75%).
- 1550nm-Sapphire-NoAperture-Energy_VS_R2.dat (Experimental data of squared crater radius (second column) produced with different irradiation energies (first column) in a sapphire sample. Irradiation wavelength 1550 nm. No aperture: *P _T_* = 100%)
- 1550nm-Sapphire-Aperture-Energy_VS_R2.dat (Experimental data of squared crater radius (second column) produced with different irradiation energies (first column) in a sapphire sample. Irradiation wavelength 1550 nm. Aperture: *P _T_* = 75%).
- 1550nm-FusedSilica-Aperture-Energy_VS_R2.dat (Experimental data of squared crater radius (second column) produced with different irradiation energies (first column) in a fused silica sample. Irradiation wavelength 1550 nm. Aperture: *P _T_* = 75%).
- 1030nm-beamImage-NoPinhole-MOx100.tif (Beam image of the profile represented in Figure 4 (b). Obtained by using a x100 microscope objective).
- 1550nm-beamImage-NoPinhole.tif (Beam image of the profile represented in Figure 5 (b). Obtained by using a x100 microscope objective).
- 1550nm-beamImage-Pinhole_MOx50.tif (Beam image of the profile represented in Figure 5 (c). Obtained by using a x50 microscope objective).
- 1550nm-beamImage-Pinhole_MOx50_crop120um.tif (120 *μm* × 120 *μm* crop of image 1550nm-beamImage-Pinhole_MOx50.tif, corresponding to the analyzed part of the image for obtaining the EBA value expressed on section 5C).
- info-images.txt (Information for the spatial calibration of beam images).

Data are available under the terms of the Creative Commons Attribution 4.0 International license (CC-BY 4.0).
