# Supplementary material for: Simple and robust method for determination of laser fluence thresholds for material modifications: an extension of Liu’s approach to imperfect beams
Source: Open Res Eur. 2021 Jun 25;1:7. Originally published 2021 Mar 24. [Version 2] doi: 10.12688/openreseurope.13073.2 (PMC10445842; doi:10.12688/openreseurope.13073.2)
Supplement: Peer review history

# Peer review: Reviewer response for version 2

**DOI:** 10.21956/openreseurope.14924.r27156

**Published:** 2021-06-30

**Authors:** Laurent Lamaignere

**Affiliations:** 1 CEA-CESTA, Le Barp, France

The authors took the remarks into account and responded to the various questions: they responded appropriately to the concerns raised in the peer review reports. The article is clear and of good quality, it sheds valuable light to the scientific community.

Is the rationale for developing the new method (or application) clearly explained?

Yes

Is the description of the method technically sound?

Yes

Are the conclusions about the method and its performance adequately supported by the findings presented in the article?

Yes

If any results are presented, are all the source data underlying the results available to ensure full reproducibility?

Yes

Are sufficient details provided to allow replication of the method development and its use by others?

Yes

Reviewer Expertise:

Laser damage metrology of optical components.

I confirm that I have read this submission and believe that I have an appropriate level of expertise to confirm that it is of an acceptable scientific standard.

# Peer review: Reviewer response for version 1

**DOI:** 10.21956/openreseurope.14143.r26678

**Published:** 2021-06-10

**Authors:** George Tsibidis

**Affiliations:** 1 Institute of Electronic Structure and Laser (IESL), Foundation for Research and Technology (FORTH), Heraklion, Greece

The submitted work represents an interesting investigation towards the evaluation of the damage threshold for material modification which constitutes a critical parameter in laser-based processing. The authors of the manuscript follow a rigorous approach to extend the method used for Gaussian beams to cases where other laser beam profiles are employed (Airy beams). They derive a solution based on analytical expressions for the radial distribution of fluence while theoretical results are tested against experimental observations. Both the predictions, results and approach constitutes a novel technique that can benefit researchers in the field of laser processing. Thus, I would recommend this work for indexing. My only concern that needs to be addressed a bit in more detail (to be elaborated further) is the concluding remark (paragraph just before the 'Conclusions') of the limitations of the technique and more specifically the range of laser energies in which the method works. Also whether the method is material and also laser wavelength-dependent.

Is the rationale for developing the new method (or application) clearly explained?

Yes

Is the description of the method technically sound?

Yes

Are the conclusions about the method and its performance adequately supported by the findings presented in the article?

Yes

If any results are presented, are all the source data underlying the results available to ensure full reproducibility?

Partly

Are sufficient details provided to allow replication of the method development and its use by others?

Yes

Reviewer Expertise:

Laser Matter Interaction, theoretical modelling of ultrafast dynamics and surface pattern formation.

I confirm that I have read this submission and believe that I have an appropriate level of expertise to confirm that it is of an acceptable scientific standard.

# Response

**Published:** 2021-06-17

**Authors:** Mario Garcia-Lechuga

**Affiliations:** Autonomous University of Madrid, Spain

Dear Dr. Tsibidis, we appreciate your positive evaluation. We address your comments on the following paragraphs. We thank you for your time and these exchanges helping us to improve our paper.   Regarding your concerns, on the new version we expand on those aspects on section 5D. The title of the section is changed into “Relevance, precision and recommendations”. We have transformed a paragraph related to the precision aspects into a list, including a new point commenting on the remaining uncertainties in the analysis. We also include some technical recommendations about the level of truncation for the potential users of the method.   Finally, the methodology is material and wavelength independent, since this methodology relies on the proven deterministic material response when subjected to ultrashort pulses. As long as the hypothesis of a strict threshold response making that material transformations are directs imprints of the laser profile the method is applicable. This holds for femtosecond laser ablation of dielectrics independently of the material band gap and the irradiation wavelength, as demonstrated in ref. 13.

# Peer review: Reviewer response for version 1

**DOI:** 10.21956/openreseurope.14143.r26553

**Published:** 2021-05-17

**Authors:** Andrius Melninkaitis

**Affiliations:** 1 Laser Research Center, Vilnius University, Vilnius, Lithuania

The paper of Mario Garcia-Lechuga and David Grojo entitled “Simple and robust method for determination of laser fluence thresholds for material modifications: an extension of Liu’s approach to imperfect beams” is devoted to an old problem, namely reproducibility of determination for material’s modification threshold and accuracy of beam radius characterization.

 In general, I enjoyed reading it: the style of writing and clarity of investigated approach is very acceptable.

 Below are my (minor) concerns related to the paper:

1. The definition of “integrated spatial distribution ( *ISD*)” perhaps should be replaced with the term “effective area” as it seems to have a physical meaning and has a dimension of area (square meters or square centimeters). Accordingly, ISD is not correctly used in Figure 1 by stating “both having an identical integrated pulse energy (ISD).” ISD – is not energy it is an area, however, it is easy to fix this discrepancy.
2. On “generality of these calculations that only depend on the filling parameter *β _G_*.”: in the present study it is assumed that aperture blocking would in all cases result in perfect airy beams (both software simulation and Pharos laser have relatively good M2 if not perfect), however, it is not clear if this assumption holds for more complicated initial beams that authors are targeting. For example: what would happen if the beam incident to the aperture is non-Gaussian or Gaussian, but has very poor M2 performance? Would the same theory work as well or also other corrections would be needed? Is “PSFLab software” able to help validate that assumption? If not then what are the limits of the presently discussed approach concerning the initial beam?
3. In the present research image analysis was used for exemplification of improvement ideas and ISD calculation. In practice, ISD could be very much affected by the camera noise and software aperture used for ISD. Two questions related to this context:
  1. Could authors compare diameter calculated from ISD and estimated experimentally by using Liu’s or their original approach?
  2. What recommendations authors could give to the integration area for measurement of energy when dealing with Airy functions: by how much energy/power detector area must be larger than the central peak diameter of Airy disk?   
4. Where exactly aperture must be placed?
5. What is the recommendation for the assessment of correction factors when using truncated beams (for any beam: is it possible to calculate those for unknown truncated input beams in the wavelength range where CCD censors do not work)?

Otherwise, the paper is very good. I would support its indexing.

 Finally, the reviewer would like to thank both authors and the editorial team for their patience while waiting for a response (it was unusually long due to a very busy schedule).

Is the rationale for developing the new method (or application) clearly explained?

Yes

Is the description of the method technically sound?

Partly

Are the conclusions about the method and its performance adequately supported by the findings presented in the article?

Partly

If any results are presented, are all the source data underlying the results available to ensure full reproducibility?

Yes

Are sufficient details provided to allow replication of the method development and its use by others?

Yes

Reviewer Expertise:

Laser damage testing, beam characterization, optics, laser physics

I confirm that I have read this submission and believe that I have an appropriate level of expertise to confirm that it is of an acceptable scientific standard, however I have significant reservations, as outlined above.

# Response

**Published:** 2021-06-17

**Authors:** Mario Garcia-Lechuga

**Affiliations:** Autonomous University of Madrid, Spain

Dear Dr. Melninkaitis, we really appreciate your overall positive evaluation and the constructive questions of your report. We address in detail your comments on the following paragraphs. We thank you for your time and these exchanges that are surely leading to an improved paper.  

**Answer to question 1.** We have converted the previous named ISD-integrated spatial distribution to the most common term (as you and the other reviewer referred) EBA-efficient beam area. This notation change is introduced throughout all the document. Additionally, we have corrected the mistake you mentioned about Figure 1.  

**Answer to question 2.** We consider that we provide a demonstration of validity of this methodology for a non-Gaussian beam on the experiment performed at 1550 nm (section 5C). On that case, by performing a truncation equal to *P _T_* = 75% we observed that the truncated function at the focal position corresponds well with the theoretical truncated function expected when illuminating the aperture with a perfect Gaussian beam (paragraph after figure 5). Therefore, as discussed on the first version, we consider that *P _T_* = 75% is an appropriate level of truncation for pulses showing a pedestal. For more irregular beams we propose the use of strong truncation ( *P _T_* < 60%), since the impact of the incoming beam shape on the spot size at the focal position will become less relevant as far as the aperture used becomes smaller. We acknowledge that it is not possible to qualify the level and nature of beam imperfections but this must be taken by the user of the method. Higher is the uncertainty on the beam or the imperfection smaller should be the aperture. Obviously, a limit will be given by the available laser power but very small aperture making possible a uniform plane wave transmitted must lead in theory to the most appropriate condition for applicability of the method In summary, even if we cannot calculate diverse beam shape with PSFlab software (the shape of the beam illuminating the aperture is always considered to be Gaussian), our proposed methodology relies more on the effect of introducing an aperture than on the non-Gaussian incoming beam. Obviously, this is relevant for common laser beam shapes, but hardly applicable to some exotic as higher-order Gaussian beams with donut profiles. This is the reason why we use term ‘imperfect’ to refer to beams with some uncertainties. Finally, we would like to point out that by introducing the aperture we are obtaining a worse M ^2^, since we are transforming expressly the beam to a non-Gaussian beam (Airy-beam like). So, in this methodology the M ^2^ is a concept to avoid. We thank you again for giving us the possibility to clarify here these important aspects for the reader.

**Answer to question 3.** First, we agree with your comment that experimental images of the beam should be treated correctly, otherwise the efficient beam area obtained can be totally biased by the noise. For minimizing this effect, after recording the image of the beam we recorded under the same conditions (same exposure time) another image by blocking the laser beam. The “background” image is subtracted on the beam image, and those images are the ones presented on the article and on the raw data provided. It should be noticed that no negative values appear, since we kept the resulting image as a 16-bit image (positive integer values) with intensity levels from 0 to 65535. However, we agree that this treatment, as well as the difficulties of perfectly beam recording (alignment and spatial calibration of the imaging system), makes not the process of obtaining the EBA (effective beam area, previously named ISD) free of incertitude. Therefore, and responding first to the second question (3.2), our recommendation with the proposed methodology is to avoid imaging. Our methodology aims to provide a simple solution in which imaging becomes unnecessary, since it relies on the diffractive pattern resulting from introducing a circular aperture. Regarding the first question (3.1), we apologize in advance since we are not sure if we fully understand it. The calculation of a beam waist from the calculated ISD (now named EBA) applicable for a general case (any beam shape) can be a subject of debate and we do not see clearly the relevance of comparisons on this aspect. As a comment, we consider the beam diameter can be only unambiguously calculated from the EBA if the beam profile is Gaussian, since the formula relating both of them are known and simple ( *EBA* *=* *π* *w* *_0_^2^*  / 2). However, generally speaking, we do not recommend extracting the beam diameter from the EBA. About the comparison of the “real beam” (the one image) and the equivalent gaussian beam retrieved with the Liu’s method, on section 5B, we compared on the first version of the article the FWHM values obtained by imaging the beam and by performing the Liu’s method. We find an excellent agreement between the two values, with a difference of less than a 2%.

**Answer to question 4.** The aperture should be placed as close as possible to the lens. On the calculations with PSFLab, this parameter is introduced as the pupil aperture of the focusing lens. We thank you to help us to clarify this very important practical aspect. This information is now added in the text (5A) of the new version.  

**Answer to question 5.** On section 5D, now renamed it as “Relevance, precision and recommendations” we have included a new paragraph: Finally, as recommendations for the level of truncation to be applied, we have observed that *P _T_* = 75% is an appropriate level when having a pseudo-Gaussian beam with a pedestal. In the case of more irregular beams or totally unknown beams, we propose to perform experiments under two levels of truncation, one with moderate truncation ( *≥* *P* *T*= 75% *)*   and another with strong truncation ( *P* *T* *≤*  60%). Obtaining similar fluence values under the two experiments would indicate that a moderate truncation level is sufficient to transform the beam into an Airy-like beam. Otherwise, the most accurate fluence threshold value is obtained under the strongest truncation, since strong spatial filtering reduces the influence of irregularities of the input beam.  “

# Peer review: Reviewer response for version 1

**DOI:** 10.21956/openreseurope.14143.r26555

**Published:** 2021-04-29

**Authors:** Laurent Lamaignere

**Affiliations:** 1 CEA-CESTA, Le Barp, France

Review on the paper “Simple and robust method for determination of laser fluence thresholds for material modifications: an extension of Liu’s approach to imperfect beams” by Mario Garcia-Lechuga and David Grojo.

 I really like the idea of ​​a paper that focuses on the accuracy and the precision of the threshold measurements made. In some cases where it is difficult to know the energy distribution in the laser beams, these are supposed to be "perfectly" Gaussian at the focus of a lens. This is not always strictly the case, and the small deviation from this assumption can lead to large errors when Liu's method is used to determine the thresholds (ablation and/or damage thresholds) of a material or even the diameter of a beam. Therefore, the authors had the clever idea of ​​rendering the beam of perfectly known shape using diaphragms to obtain an Airy spot on the component, a spot of known and mastered shape. This makes sense and the theoretical proof is supported by experimental validation. The authors propose a smart method with the keys to put it into practice and use it with relevance. The two aspects of the article, theory and experience, make it compelling and interesting. I therefore recommend this article for indexing and I also recommend that readers consider the tips and guidelines reported in this article. Some points need to be clarified and improved before indexing.

 Few main comments:

- Accuracy of the method – error bars

The first point deals with the accuracy of the method: the purpose of this paper is to obtain much more accurate thresholds thanks to the shaping of beams of perfectly known shape. Very good. It is therefore even more imperative to discuss the correctness of the method, its precision, its intrinsic precision as well as the gain compared to measurements obtained with imperfectly known beams. It seems to me that this point is not discussed. The article would be even more relevant if the reader could actually see the contribution of this experimental approach. In other words, error bars are not discussed with this approach. The paper would be improved with such a discussion.

- Vocabulary and notations:

The second point deals with the vocabulary and notations:

 Wouldn’t it be as simple to write that w _0_ is the beam radius at 1/e², instead of 2w _0_ is the beam diameter at 1/e²?

 Also, in Eq. 8, w _Airy_ is the radial waist at 1/e². OK. In that case, report w _0_ as the beam radius in Eq. 1. Otherwise, it is confusing.

 ISD (integrated spatial distribution) is also named *equivalent area* in the laser community. This equivalent area (ISD) is therefore the area at 1/e of the peak fluence F _0_. In that case, it would certainly be as simple to report all the equations of section 2 at 1/e instead at 1/e²!

 Eq. 8: I didn’t catch why r’=r*2.5838/w _airy_, why **2.5838**? Is it P/1.22?

- Limitation of the method:

The third point is about one limitation of the method that I would like the authors to make readers aware of: by definition the energy threshold of the material studied is unknown. Therefore how can the users choose with certainty the correction factors given in Table 1. These factors are given for different maximum energies above energy threshold. It is therefore not trivial to select the right one.

- Comparison with literature:

The last point deals with the discussion about results in this paper and results from the literature: The comparison with Winkler data is probably not appropriate. The agreement between thresholds could be fortuitous considering first that fused silica samples are different but more than this considering that wavelengths are different (1030 nm in this work versus 800 nm in the Winkler’s paper). The threshold dependence with wavelengths is reported in reference 5 of this paper [Gallais, 2015]. It is also the case in this paper where it is reported that thresholds at 1550nm and at 1030nm are different for fused silica. Therefore, is there a reason to obtain the same thresholds at 1030 and 800 nm for fused silica? Considering again it could be fortuitous, I suggest avoiding such a comparison.

 Other minor points that could be taken into account:

- Title: which beams are considered imperfect? The Airy beams? One might think subsequently that the Airy beams are imperfect! I am not comfortable with the term ‘imperfect’. I do not know what an imperfect beam is. More it could be an imperfection in the time domain! I suggest the authors to propose a more precise title.
- Table 2 should arrive before Figure 3.
- Is caption of Figure 3 right when reporting Equation 12?
- Could the authors comment the fact on figure 3(a) that the correction factors are higher and smaller than the unity for a given P _T _(for example P _T_=0.75 or 0.60)?
- In the abstract, the notion of ‘spatial’ should be added: ‘…an ideal Gaussian spatial profile…’ to avoid confusion with ‘temporal profile’, which is also another topic that could be addressed.
- Figure 2: it is difficult to distinguish the color of the different beam profiles.
- Page 9, last paragraph: it is Figure 4(a) and not Figure 3(a)? Right ?

Is the rationale for developing the new method (or application) clearly explained?

Yes

Is the description of the method technically sound?

Yes

Are the conclusions about the method and its performance adequately supported by the findings presented in the article?

Yes

If any results are presented, are all the source data underlying the results available to ensure full reproducibility?

Yes

Are sufficient details provided to allow replication of the method development and its use by others?

Yes

Reviewer Expertise:

Laser damage metrology of optical components.

I confirm that I have read this submission and believe that I have an appropriate level of expertise to confirm that it is of an acceptable scientific standard, however I have significant reservations, as outlined above.

# Response

**Published:** 2021-06-17

**Authors:** Mario Garcia-Lechuga

**Affiliations:** Autonomous University of Madrid, Spain

Dear Dr. Lamaignere, we were pleased to read your overall positive evaluation. We address your questions in details in the following paragraphs. We thank you for your time and comments that are surely enriching the content of our paper.

**Answer to “Accuracy of the method – error bars”** It is hard to make a general statement on the gain in precision as the gain depends on the beam imperfections that can take various forms. Overall, the reader will understand that stronger are the imperfections, higher is the benefit. To better convey this message and to address your comment with more quantitative evaluation of the precision, we have added information in the specific experimental case of our report. In the new version of  section 5B and 5C, we now systematically compare (in percentage) the values obtained under the different methodologies. In particular, on the third paragraph of section 5C, we have modified a phrase to better illustrate the enormous error when applying Liu’s method to not-Gaussian (or not-Airy) beams: “This illustrates the enormous fluence determination error (>60 %) when directly applying the Liu’s method for our OPA imperfect beam” Additionally, we expand on these aspects in the section 5D. The title of the section is changed into “Relevance, precision and recommendations”. We have  transformed a the paragraph in a list, including a point commenting on the remaining uncertainties in the analysis: "It is estimated, if the other considerations are fulfilled, that the obtained fluence value should not be expressed with a better accuracy of ±5 %. This percentage is a conservative estimation accounting for the following aspects: possible uncertainties on selecting the most appropriate correction factors (especially when extrapolations of theoretical calculations of Figure 3 are needed to describe the practical case) or particular material responses interfering on the measured ablated area (as for example the melted rims formed in some glasses, ref [9, 13]). On this second aspect,  imprecisions due to subjective ablated area determination are not considered here, since in our metrology is based on user-independent analysis as referred on a previous publication [ref. 29].”

**Answer to “Vocabulary and notations”** Following your request, we have clarified that w0 refers to the Gaussian beam radius in the new version of the paper. We thank you and second referee for making us aware of the ‘efficient beam area’ as a more common terminology in the laser community. To avoid any confusion, we have changed our term “ISD-integrated spatial distribution” into “EBA-efficient beam area”. This notation change is introduced throughout all the document. On the other hand, we do not really understand the comment “This equivalent area (ISD) is therefore the area at 1/e of the peak fluence F0. In that case, it would certainly be as simple to report all the equations of section 2 at 1/e instead at 1/e²!”. In our view, following the mathematical description expressed in eq 4. the EBA (previously named as ISD) is totally independent of any definition with 1/e or  1/e², since it is based on a  2D integration of all the space (-infAiry, why 2.5838? Is it P/1.22?” we apologize for having introduced this apparently “weird” number without any context. This value is directly coming from the mathematical definition of the Airy function. According to a normalized Airy function ( *g(x) = [2·J _1_(x)/x]^2 *), the value corresponding to *g(x _1_) =*1/e² (0.1353…) is found at a position equal to x _1_=2.5838... Therefore, for the easiness of definition of an Airy function with a desired waist ( *w*_*_Airy_*_ *)* , it is needed to make a change of variable according to this number. This corresponds to the expression introduced on Eq. 8. Small changes have been done to clarify this originates from a “change of variable”.

**Answer to “Limitation of the method”** First, we fully agree on your comment on the difficulties on objectively determining a threshold, by nature unknown with a beam that can also be only partly known. It can be also particularly difficult to evaluate whether there is or there is not damage when irradiating with pulse energies very close to the ablation threshold. However, our proposed methodology for determining the energy threshold is not based on 1-on-1 test strategy but in an extrapolation based on the deterministic material response with ultrashort pulses, which is the basis of Liu’s method.  So, what is referred on Table 1 are the energy threshold values obtained after applying the Liu’s method to beams that are not strictly Gaussians. Therefore, when applying a linear regression onto a non-Gaussian beams (not fully linear on the representation D ^2^ vs ln(E) ) leads to a slightly incorrect determination of the energy threshold . This deviation becomes larger when including into the linear fitting modifications produces with high excitation energies (as represented on Table 1).   So, we consider that users of this proposed methodology are not requested to make any assumption on the values of the energy threshold, the correction factors being applied afterwards. Users only need to know two things for using the corrections factors expressed on figure: the aperture transmission and the maximum excitation energy above the energy threshold of modification. This second parameter is *a priori* unknown, but the biased retrieval of the energy threshold by the linear fitting has no difficulty and can be safely used for this matter (maximum considered energy / retrieved energy threshold). We thank you for giving the opportunity to clarify here this practical aspect for the reader.

**Answer to “Comparison with literature”** We agree that this comparison is not fully necessary and can be considered inappropriate for our ‘method paper’. Our aim was only to confirm that our obtained results are somehow consistent with other reports. On the paper of Gallais et al. [ref. 5] there is a important figure (fig.5) reviewing the fluence threshold values on fused silica (800 nm and 1030 nm) reported by different authors. We initially wanted to center our discussion based on this figure. Instead, due to the complexity of comparisons, we decided to focus only on the result obtained by Winkler et al. [ref. 32], since the irradiation conditions were the closest to the ones we have used (in particular pulse duration of 180 fs). In order to clarify this aspect, we have introduced some changes in the text referring to this figure but also highlighting the importance to be very cautious with comparisons. We can also comment here on the wavelength dependence. We can already confirm that the difference on fluence threshold values in dielectrics on the near-infrared range (>800 nm) are practically wavelength independent, at least at the pulse duration we performed the experiments (around 200 fs). This interesting response analysis made possible with the method that we introduce in the present paper will be the subject of another publication under preparation.

**Answer to “Other minor points that could be taken into account”** **Answer point 1. **We have carefully considered the possible change in the title of “imperfect beams” into “non-Gaussian beams”. We acknowledge that the term “imperfect” may sound less precise but we believe it better conveys our message. This is because our method does not only treat deviation from a Gaussian hypothesis but can be in principle applied for any imperfect or imperfectly known beam. With other words, a perfectly known beam even non-Gaussian can be considered as ideal beam for a threshold determination without our method.

**Answer point 2. **“Table 2 should arrive before Figure 3”. We agree (and it was like that on our submitted word document). We will do our best to ask to the editorial team to introduce this change on the new published version.

**Answer point 3. **You are totally right. There is a displacement on the equation numbering that is corrected in the new version. For (a) should be eq.10 and for (b) should be eq. 11.

**Answer point 4. **We agree that those correction factors are not totally intuitive. The explanation of having a correction factor higher than 1 means that the equivalent Gaussian beam (what the Liu’s plot provides us) exhibits a larger EBA than the truncated beam. This occurs when selecting only irradiations at moderate excitation levels (e.g. 2 times the E_th), which is equivalent to thresholding only the top part of the truncated beam (similar shape than a Gaussian beam) but not the lower part (narrower than a Gaussian beam). However, as shown in table 1 on the particular case of doing this procedure on a perfect Airy beam (P _T_ ideally equal to 0) fluence correction factors are always smaller than 1. On that case, even if the area of the central lobe is overestimated when accounting the equivalent Gaussian beam (Liu’s plot), the total EBA is underestimated because of the contribution of the rings of the Airy beam (which are inexistant on the retrieved equivalent Gaussian beam). So, finally, addressing the particular truncation you mention (P _T_=0.75 or 0.60), and given the fact that in these cases the contribution of the surrounding rings to the total EBA is less pronounced than for a perfect Airy beam, it remains totally possible to obtain fluence correction factors above or below 1 depending on the maximum considered energy accounted for the linear fitting process. We thank you again for giving us the possibility to clarify also this important aspect for the reader.

**Answer point 5. **We included on the abstract the notion of spatial. (“spatial Gaussian beam”).

**Answer point 6. **The colors are corrected on the new version. We have followed the same color criteria than the one in Figure 3.

**Answer point 7. **You are totally right. On the last paragraph of Page 9 we should have referred to Figure 4(a). We introduce this change on the new version.
